# Correlation Between the Clinical Diagnosis of Bacterial Vaginosis and the Results of a Proline Aminopeptidase Assay

**DOI:** 10.1155/S1064744994000037

**Published:** 1994

**Authors:** George H. Nelson, Janice L. Bacon

**Affiliations:** 1 Department of Obstetrics and Gynecology University of South Carolina School of Medicine Columbia SC USA; 2 Two Richland Medical Park Suite 208 Columbia SC 29203 USA

## Abstract

*Objective:* The object of this study was to develop a simple and inexpensive test for detection of bacterial vaginosis (BV) in pregnant patients and to test its accuracy in a clinic population.

*Methods:* We developed a modified proline aminopeptidase (PAMP) assay to detect BV and compared the results of the assay with the clinical diagnosis of BV.

*Results:* The results of the PAMP assay in 55 asymptomatic and 50 symptomatic subjects significantly correlated with a clinical diagnosis of BV. The prevalence of BV in the asymptomatic population was 42% (PAMP assay) and 38% (clinical diagnosis). In the symptomatic population, it was 50% (PAMP assay) and 54% (clinical diagnosis). The sensitivity, specificity, accuracy, positive predictive value (PPV), and negative predictive value (NPV) of the PAMP assay were 86, 85, 86, 78, and 91%, respectively, in asymptomatic patients and 89, 96, 92, 96, and 88%, respectively, in symptomatic patients.

*Conclusions:* The modified PAMP assay, which we describe, met our goals for simplicity, cost, and accuracy. We feel it could be best used as a screening test for BV in asymptomatic pregnant patients.


Infectious Diseases in Obstetrics and Gynecology 1:173–176 (1994)

© 1994 Wiley-Liss, Inc.

### Correlation Between the Clinical Diagnosis of Bacterial Vaginosis and the Results of a Proline Aminopeptidase Assay

George H. Nelson and Janice L. Bacon
*Department of Obstetrics and Gynecology, University of South Carolina School of Medicine, Columbia, SC*

ABSTRACT

***Objective:*** **The object of this study was to develop a simple and inexpensive test for detection of bacterial vaginosis (BV) in pregnant patients and to test its accuracy in a clinic population.**

***Methods:*** **We developed a modified proline aminopeptidase (PAMP) assay to detect BV and compared the results of the assay with the clinical diagnosis of BV.**

***Results:*** **The results of the PAMP assay in 55 asymptomatic and 50 symptomatic subjects significantly correlated with a clinical diagnosis of BV. The prevalence of BV in the asymptomatic population was 42% (PAMP assay) and 38% (clinical diagnosis). In the symptomatic population, it was 50% (PAMP assay) and 54% (clinical diagnosis). The sensitivity, specificity, accuracy, positive predictive value (PPV), and negative predictive value (NPV) of the PAMP assay were 86, 85, 86, 78, and 91%, respectively, in asymptomatic patients and 89, 96, 92, 96, and 88%, respectively, in symptomatic patients.**

***Conclusions:*** **The modified PAMP assay, which we describe, met our goals for simplicity, cost, and accuracy. We feel it could be best used as a screening test for BV in asymptomatic pregnant patients.**
**© 1994 Wiley-Liss, Inc.**

KEY WORDS
Enzyme method, pregnancy, vaginal infections

Because of growing evidence[1–3] that bacterial vaginosis (BV) may be a contributing factor in preterm labor and delivery, a simple method for detection of BV is highly desirable. Schoonmaker et al.[4] recently described a new method for the diagnosis of BV using a proline aminopeptidase (PAMP) assay. They compared the Gram stain with 2 PAMP assays using either L-proline B-naphthyamide or L-proline P-nitroanalide as substrate in the diagnosis of BV. We have modified the proline nitroanalide assay and report here the results of this modified assay in the diagnosis of BV in asymptomatic and symptomatic pregnant patients. We chose the proline nitroanalide substrate because proline naphthyamide yields a carcinogenic end product.

### SUBJECTS AND METHODS

Patients attended either the Teen or OB Clinic at Richland Memorial Hospital. All patients seen in these clinics were under the supervision of the author (J.L.B.) who made the clinical diagnoses. A clinical diagnosis of BV, *Trichomonas* vaginitis (TV), a yeast vaginitis (YV), or no vaginitis (NV) was made using the following criteria: BV (3 or more of the following: presence of 20% or more

**Address correspondence/reprint requests to Dr. George H. Nelson, Two Richland Medical Park, Suite 208, Columbia, SC 29203.**

Received July 12, 1993
Accepted January 5, 1994

clue cells, fishy odor with KOH, vaginal pH > 4.5, or thin gray-white discharge in patients who were diagnosed with BV alone; 2 or more of the following: presence of clue cells, fishy odor with KOH, or vaginal pH > 4.5 in patients with mixed infections); TV, presence of trichomonads on wet mount; YV, presence of hyphae on wet mount; and NV, absence of any diagnosis above. All study patients were tested for BV, TV, and YV.

The PAMP assay was done in the following manner. Vaginal secretions were taken with a cotton-tipped applicator and placed in a 1.5 ml plastic centrifuge tube containing 1.0 ml of normal saline. The applicator tip was broken off and the tube was sealed with a plastic cap which comes attached to the tube. The tubes were placed in a refrigerator and were assayed within 3 days of collection. The applicator tip was removed from the tube with tweezers and the secretions were extracted into the saline using a gloved hand.

The tubes were centrifuged for 5 min at 10,000*g* in a Fisher Scientific Microcentrifuge, Model 50-A (Fair Lawn, NJ). The supernatant was decanted and the following were added to the pellet in each tube: 50 μl solution 1 (0.05 M Trizma, T-4003, pH 7.4) and 100 μl solution 2 (2 mg/ml of L-proline p-nitroanalide, p-5267, made up in solution 1). A blank and a standard tube were run with each batch of sample tubes. The blank tube contained 100 μl each of solutions 1 and 2. The standard tube contained 100 μl solution (containing 20 mU of microsomal leucine aminopeptidase, L-5006, made up in solution 1) and 100 μl solution 2. All chemicals were purchased from Sigma Chemical Company (St. Louis, MO). The reason 100 μl of solution 1 (blank tube) and 100 μl of enzyme (standard tube) are added instead of 50 μl is because the volume of the pellet plus fluid remaining in the sample tube after centrifugation and decantation is about 50 μl. Therefore, all tubes contained approximately 200 μl total volume for color development. Leucine aminopeptidase was used as a standard because PAMP is not available commercially and leucine aminopeptidase cleaves the proline from proline nitroanalide in a similar manner as PAMP. After the preparation of the tubes (one or more samples, one blank, and one standard), they were incubated in a Dubnoff metabolic shaking incubator (Fisher Scientific, Norcross, GA) at 37°C for 1 h. The samples were then removed, and the depth of the yellow color was compared visually to the standard and blank tubes. A positive test in a sample tube is a depth of color ≥ the color in the standard tube.

This method differs from the original method described by Schoonmaker et al.[4] in the following ways:

1. Sample collection, color development, and evaluation take place in the same tube. The original method resuspends the cell pellet and transfers it to a microtiter plate well.
2. A water incubator is used instead of an air incubator.
3. Incubation time is reduced from 4 h to 1 h.
4. A standard tube is used for comparison to the sample tube. The original article states: "A yellow color indicated a diagnosis of bacterial vaginosis; a clear color was scored as negative for bacterial vaginosis."[4]

Statistical analyses were done using the $\chi^2$ test for independence. $P < 0.05$ was considered significant.

### RESULTS

All patients were pregnant at the time of the assay with gestational ages ranging from first trimester to 41 weeks gestation. No attempt was made to control for patient age or gestational age. Patients were asked if they had any complaints of vaginal burning or itching or if they had an abnormal vaginal discharge. Those who responded negatively were listed as asymptomatic, while those who answered affirmatively were listed as symptomatic.

A comparison of the results of the PAMP assay with the clinical diagnoses of 55 asymptomatic pregnant patients is shown in Table 1. As can be seen, there was a highly significant correlation between the clinical diagnosis of BV and the PAMP assay. Likewise, Table 2 illustrates a highly significant correlation between the clinical diagnosis of BV and the PAMP assay in symptomatic patients.

Of the 105 subjects studied, 41 (39%) had BV alone, 6 (5.7%) had BV plus TV, 1 (1%) had BV plus TV plus YV, 5 (4.8%) had TV alone, 9 (8.6%) had YV alone, and 1 (1%) had TV plus YV. Therefore, in this population, 8 (7.6%) had mixed infections. Interestingly, all mixed infections were found in symptomatic patients.

**TABLE 1. Correlation between the clinical diagnosis of BV, NV, TV, or YV and the PAMP assay in asymptomatic pregnant patients**

| PAMP assay | Clinical diagnosis (no. of patients) BV positive* | | BV negative* | |
|---|---|---|---|---|
| Postive | 18 | | 5 | |
| Negative | 3 | | 29 | |
| | BV alone | NV | TV | YV |
| Positive | 18 | 4 | 1 | 0 |
| Negative | 3 | 25 | 2 | 2 |

*N = 55; $\chi^2 = 26$; $P < 0.001$.

**TABLE 2. Correlation between the clinical diagnosis of BV, NV, TV, or YV and the PAMP assay in symptomatic pregnant patients**

| PAMP assay | Clinical diagnosis (no. of patients) BV positive* | | | BV negative* | | | |
|---|---|---|---|---|---|---|---|
| Positive | 24 | | | 1 | | | |
| Negative | 3 | | | 22 | | | |
| | BV alone | BV + TV | BV + TV + YV | NV | TV | YV | TV + YV |
| Positive | 18 | 5 | 1 | 0 | 1 | 0 | 0 |
| Negative | 2 | 1 | 0 | 13 | 1 | 7 | 1 |

*N = 50; $\chi^2 = 35$; $P < 0.001$.

**TABLE 3. Sensitivity (Sen), specificity (Spec), accuracy (Acc), PPV, and NPV of the PAMP assay in asymptomatic and symptomatic pregnant patients[a]**

| | Asymptomatic | Symptomatic |
|---|---|---|
| Sen (%) | 86 | 89 |
| Spec (%) | 85 | 96 |
| Acc (%) | 85 | 92 |
| PPV (%) | 78 | 96 |
| NPV (%) | 91 | 88 |

[a]Sen = true positive (TP) divided by TP + false negative (FN); Spec = true negative (TN) divided by TN + false positive (FP); Acc = TP + TN divided by TP + TN + FP + FN; PPV = TP divided by TP + FP; NPV = TN divided by TN + FN.

Table 3 illustrates the sensitivity, specificity, accuracy, positive predictive value (PPV), and negative predictive value (NPV) of the PAMP assay.

### DISCUSSION

When this study was established, our intention was to set up a PAMP assay that was easy, inexpensive, required no sophisticated equipment, demanded minimum technical expertise, and was reasonably accurate. The procedure described by Schoonmaker et al.[4] seemed to serve as an excellent starting point. The modified procedure as described here required taking a specimen of vaginal fluid with a cotton swab, extracting the fluid into saline in a centrifuge tube, a 5 min centrifugation, decantation, making 1 measurement each of 50 μl and 100 μl, a 1 h incubation in an ordinary water bath, and visually reading a positive or negative test comparing the sample to a standard.

In our study, the sensitivity of the PAMP assay was 88% overall, which is comparable to that reported for this assay by Shoonmaker et al.[4] (93%) and Thomason et al.[5] (81%). Shoonmaker et al.[4] compared the PAMP assay with the Gram stain, while Thomason et al.[5] compared the assay with the clinical diagnosis of BV.

The data reported in Table 3 assume that the clinical diagnosis of BV is correct in every instance. Since the clinical diagnosis of BV is based on subjective evaluation, it is entirely possible that the false-positive and the false-negative measurements reported for the PAMP assay may not be truly false-positive and false-negative values. Therefore, the accuracy of the PAMP assay may actually be better than we report here.

This would seem appropriate in light of the report by Eschenbach et al.[6] They report that in 311 patients diagnosed with BV by Gram stain criteria, 29% had a homogeneous discharge, 43% had a fishy odor with KOH, 97% had a pH ≥ 4.7, 81% had the presence of clue cells, and 78% had 20% or more clue cells. However, the PPV of the assay may be lower in a population of patients with a lower prevalence of BV. Perhaps it might be more appropriate not to use terms such as sensitivity, specificity, and PPV when conducting a study such as this. Neither of the techniques used (clinical diagnosis, PAMP assay) provides a definitive diagnosis. Perhaps we should only conclude that the techniques appear to be evaluating the same process. The $\chi^2$ test for independence illustrates that the odds the 2 measurements (clinical diagnosis, PAMP assay) are totally independent of one another are less than 1 in 1,000.

While the assay could be used in a variety of settings, we feel that a definite role would be its potential use as a screening test in asymptomatic pregnant clinic patients. This may be particularly applicable to public health settings. In our study in this population, 38% (by clinical diagnosis) and 42% (by positive PAMP assay) were found to be harboring the organisms associated with BV.

While the assay described takes longer to complete than a clinical diagnosis by a physician, the assay can be performed by non-physician personnel with minimal training in laboratory medicine. In addition, it appears that the assay will detect BV in the asymptomatic patient who would not normally be checked for BV in a clinical setting. This may be particularly pertinent in view of the recent data reported by Riduan et al.[7] They showed that preterm delivery was more prevalent in patients with BV diagnosed at 16–20 weeks gestation but not in patients diagnosed with BV at 28–32 weeks gestation. Perhaps screening a large group of asymptomatic patients with the PAMP assay at 16–20 weeks gestation would yield promising results.

### REFERENCES

1. Martius J, Krohn MA, Hillier SL, et al.: Relationships of vaginal *Lactobacillus* species, cervical *Chlamydia trachomatis*, and bacterial vaginosis to preterm birth. Obstet Gynecol 71:89–95, 1988.
2. Gravett MG, Nelson HP, DeRouen T, et al.: Independent associations of bacterial vaginosis and *Chlamydia trachomatis* infection with adverse pregnancy outcome. JAMA 256: 1899–1903, 1986.
3. Gravett MG, Hummel D, Eschenbach DA, et al.: Preterm labor associated with subclinical amniotic fluid infection and with bacterial vaginosis. Obstet Gynecol 67:229–237, 1986.
4. Schoonmaker JN, Lunt BC, Lawellin DW, et al.: A new proline aminopeptidase assay for diagnosis of bacterial vaginosis. Am J Obstet Gynecol 165:737–749, 1991.
5. Thomason JL, Gelbart SM, Wilcoski LM, et al.: Proline aminopeptidase activity as a rapid diagnostic test to confirm bacterial vaginosis. Obstet Gynecol 71:607–611, 1988.
6. Eschenbach DA, Hillier S, Critchlow MS, et al.: Diagnosis and clinical manifestations of bacterial vaginosis. Am J Obstet Gynecol 158:819–828, 1988.
7. Riduan JM, Hillier SL, Utomo B, et al.: Bacterial vaginosis and prematurity in Indonesia: Association in early and late pregnancy. Am J Obstet Gynecol 169:175–178, 1993.
